# Effect of packaging materials and storage temperature on the shelf stability of *Awaze* paste

**DOI:** 10.3389/fnut.2024.1503328

**Published:** 2025-01-07

**Authors:** Biadge Kefale, Mulugeta Admasu Delele, Solomon Workneh Fanta, Solomon Abate

**Affiliations:** ^1^Faculty of Chemical and Food Engineering, Bahir Dar Institute of Technology, Bahir Dar University, Bahir Dar, Ethiopia; ^2^Ethiopian Institute of Agricultural Research, Holeta Agricultural Research Center, Food Science and Nutrition Research, Holeta, Ethiopia; ^3^Leibniz Institute of Agricultural Engineering and Bioeconomy (ATB), Potsdam, Germany; ^4^Ethiopian Institute of Agricultural Research, Head Quarter, Food Science and Nutrition Research, Addis Ababa, Ethiopia

**Keywords:** shelf life, food quality, glass bottle, food safety, *Awaze* paste

## Abstract

**Background:**

It is well known that deterioration is a big concern in the food supply chain. The problem is more serious in handling of traditional foods in developing country such as Ethiopia, due to the limited knowledge about the optimum processing, packaging and storage conditions.

**Objective:**

This study aimed to investigate the effect of packaging material and storage condition on the shelf life of Ethiopian traditional *Awaze* paste.

**Methods:**

Six types of packaging materials were employed: *Shekella* pot, *Gourd (Qelle)*, high-density polyethylene (plastic bag), plastic bottle, glass bottle and metal can. These packaging materials are traditionally used by household producers and cottage industries in Ethiopia. The paste was stored at two temperatures: room temperature (21 ± 2°C) and refrigeration temperature (4°C).

**Results:**

Physical changes, color (a) value, pH, acidity, yeast and mold levels, total bacterial count (TBC), and lactic acid bacteria count (LAB) were assessed every 60 days over a period of 300 days. pH value, acidity, yeast and mold, TBC, and LAB count were significantly (*p* < 0.05) affected by packaging material, storage temperature and storage period. After 300 days of storage, the highest yeast and mold count, 4.06 log CFU/g, was observed in samples stored in plastic bags. The highest total bacterial count (TBC), 4.12 log CFU/g, was found in samples stored in metal cans. The samples stored in glass bottles at refrigeration temperature (4°C) were found to have a color (a*) value difference of 11.5 to 13.85, a yeast and mold count value of 3.2 log cfu/g, and a TBC value of 2.97 log cfu/g, which were acceptable after 300 days of storage as per the international food standards.

**Conclusion:**

Taking into account all parameters including physical changes (color, mold growth, texture), acidity, yeast and mold, TBC, and LAB count, *Awaze* paste could be stored in glass bottles at 4°C for up to 300 days.

## Introduction

*Awaze* is a major fermented sauce used in various ways in rural Ethiopian households (1). It is created by fermenting a blend of spices such as red pepper, ginger, red onion and garlic. This mixture also includes smaller quantities of cardamom, fenugreek, white cumin, basil, black cumin, *mekelesha*, rue, coriander, rosemary, thyme, and salt. Lactic acid bacteria and Yeast are involved in the 30-day fermentation process, which occurs at ambient temperature (2). This process enhances the taste and flavor of the *Awaze* paste, making it rich in antioxidants, fiber, and minerals, with good storage stability because of the high spice and salt content. Red onion, ginger, and the wet spices (used in small amounts) are perishable, with spoilage typically caused by improper handling, natural enzymes, chemical reactions, and changes during storage. Post-harvest losses of red onion can be reduced through processing and proper storage (3), but there is a need to explore alternative preservation methods and value-added products. *Awaze* paste is a minimally processed alternative spice based food that can be stored for extended periods without significant changes in quality or freshness (4).

Fermented *Awaze* paste in Ethiopia is traditionally produced by each rural households in the winter, following the November harvesting season for red pepper. It is then consumed throughout the year by individual rural Ethiopian households (4, 5). *Awaze* paste is commonly prepared using traditional fermentation process and stored using traditional storage materials [*Shekella* pot and *Gourd (Qelle)*] at individual households. Modern industrial packaging materials such as plastics, glass and cans are not commonly used by the rural community.

Previous studies have investigated the formulation and optimization of *Awaze* paste in relation to the method of preparation and proportion of ingredients (1). Other studies have investigated the preservation of *Awaze* paste. Woldemariam et al. (6) reported on the microbial inactivation of *Awaze* paste treated by high pressure processing. Idris et al. (7) presented microbial and physicochemical studies on the fermentation of *Awaze* paste. Tigu et al. (8) reported on the probiotic properties of lactic acid isolates from *Awaz*e paste. Tsegaye et al. (2) studied the behavior of *E. coli* O157: H7 after the fermentation of *Awaze* stored at atmospheric and refrigeration temperatures. Additionally, the effect packaging and storage have been conducted on various red pepper paste products from different countries around the world. The influence of packaging films on the characteristics of *Gochujang* was studied by Lee et al. (9). The effect of packaging materials on the quality of red pepper paste was reported by Uzel et al. (10). The inactivation of *Aspergillus flavus* in pepper paste by gamma ray and X-ray was studied by Byun et al. (11). Freeze-drying of chili paste was studied by Man et al. (12). The pasteurization of red pepper paste by ohmic heating was presented by Cho et al. (13).

Recently, the processed spice market has experienced growth, largely attributed to the success of fast food chains and restaurants. In the domestic channel, final users of processed spices include food processing industries (5–10%), the retail sector (80–90%), and the catering sector (5–10%) in Ethiopia (14). However, small-scale processors often lack the technologies necessary for proper storage and preservation of *Awaze* paste. Packaged paste products, made using traditional Ethiopian household procedures, have become popular in many homes. Consumers prefer non-pasteurized paste without preservatives to maintain high sensory quality. Unfortunately, the storage and packaging conditions do not always meet the paste’s requirements, leading to deterioration in quality during storage.

Literature reviews have revealed that there is limited research on the microbial and physicochemical properties of *Awaze* during storage using various packaging materials and temperatures. In rural households in Ethiopia, traditional packaging materials such as *Shekella* pots and *Gourd (Qelle)* are commonly used to store *Awaze* paste at room temperature. In urban households in Ethiopia, there is a very limited production of *Awaze* paste and it is commonly stored using packaging materials like plastic bottles, glass bottles, and plastic bags, both at room temperature and refrigeration conditions. Nevertheless, the best packaging materials and storage conditions to prolong the shelf life of *Awaze* in Ethiopia have not known yet.

To fill this gap, this study aims to evaluate the shelf life of *Awaze* by examining how its physicochemical and microbiological properties change when it is stored in different packaging materials at various temperatures. Acquiring knowledge about the physicochemical and microbial properties such as color value (a*), physical changes, pH, acidity, yeast, mold, and total bacteria under different packaging and storage conditions is crucial for determining the effective shelf life of the paste. This information could help to recommend suitable packaging material and storage conditions to household producers, cottage industries, entrepreneurs, supermarkets, and industries.

## Materials and methods

### Collection and preparation of *Awaze* paste

In the current study, raw materials for *Awaze* paste were obtained from Bure district, Amhara region, Ethiopia in September 2022. Red pepper was sourced from there, while garlic, ginger, red onion, cardamom, white cumin, fenugreek, black cumin, basil, rue, *mekelesha*, coriander, rosemary, thyme, and salt were purchased from *Menegasha* Market in September 2022, Oromia region, Ethiopia. After purchasing, the spices were transported to Food research laboratory at Holeta Agricultural Research Center, Ethiopia for product development.

*Awaze* paste used in the study was prepared according to the method (1). An optimized formula was used for the storage experiment of *Awaze* paste. Independent variables included (65.66: 10: 19.08: 5.25% red pepper, garlic, red onion, ginger, respectively), which were mixed with controlled variables of 5 g cardamom, 5 g fenugreek, 2.5 g white cumin, 2.5 g basil, 1.25 g black cumin, 1.25 g *mekelesha*, 1 g rue, 0.5 g coriander, 0.4 g rosemary, 0.4 g thyme, and 20 g salt. The mixed ingredients were milled and 200 g of composite powder was mixed with 300 mL of boiled water in a 400 mL capacity screw cap bottle to prepare *Awaze* paste.

### Storage method of *Awaze* paste

Two traditional storage materials, *Gourd (Qelle)* and *Shekella* pots, commonly used by household producers, were employed. In Addition, modern industrial packaging materials that include high-density polyethylene plastic bags, plastic bottles, glass bottles, and metal cans were used. These packaging materials were selected based on a preliminary survey conducted in Addis Ababa on packaging materials used for packaging and storing of paste foods in super markets. Prior to filling, the storage materials were sterilized for 20 min at 120°C to eliminate microbes (15). Subsequently, 200 g of *Awaze* paste was filled into each storage material and stored at two different temperature conditions: room temperature (21°C) and refrigeration temperature (4°C) based on the environmental conditions of household producers and market utilization of the paste by consumers in Ethiopia.

### Experimental design and treatment combination

The study was conducted using a completely randomized design (CRD). The experiment utilized a three-factor factorial design. The three independent variables were packaging materials (six levels: Gourd, *Shekella* pot, plastic bag, plastic bottle, glass bottle, and metal can), storage temperature (two levels: 4°C and 22°C), and storage duration (five levels: 2, 4, 6, 8, and 10 months). Quality analysis of the stored paste samples was performed every 2 months over a period of ten months. As per the household consumption practice, the packages were opened at each sampling time and closed back as soon as the samples were taken. The initial values measured at time zero, before storage began, were used as baseline data to evaluate the quality degradation trend of the product.

### Physical change

Physical changes such as mold growth, and texture (softness) of the paste were observed (visual observation) and recorded at 60-day intervals up to 300 days.

### Color (a*) value

The color of fresh and stored *Awaze* paste samples was measured using Hunter Lab Mini Scan XE colorimeter (Hunter Associates Laboratory, Reston, United States). Prior to conducting color measurements, the Mini Scan XE colorimeter device was calibrated with white and black standard calibration plates. The color values (a*) were expressed as a* (redness/greenness) (16).

### pH value

The pH of the product was obtained following the method (17). The pH of the prepared paste was determined by mixing a 10-gram sample with 100 mL of distilled water, and then the pH was measured by dipping the calibrated electrode of a pH meter (Mettler Toledo, China) into the prepared sample solution.

### Titratable acidity

The acidity of the prepared paste was determined titrimetrically according to the method (17). A 10 g sample was added to 100 mL of deionized water, stirred gently, and the mixture was allowed to stand for 1 hour. Then, 10 mL of the solution was taken, 0.5 mL of phenolphthalein was added, and titrated with 0.1 N NaOH until a pink color appeared for 30 s.

### Microbial profile

For detecting microbial growth, standard methods were used (18). Samples (25 g) were dissolved with 225 mL of sterile water containing 0.1% peptone. Serial dilutions were performed, and the diluted samples (1 mL) were applied to the surface of the media using a pouring technique. Duplicate plates were prepared in all cases. The TBC was estimated by applying plate count agar and incubating at 30°C for 48 h. PDA was used to isolate yeast and mold, incubating at twenty five degree centigrade (25°C) for 48 h. Lactic acid bacteria (LAB) were cultured on MRS agar and incubated aerobically at 30°C for 48 h. Coliforms were estimated by pouring VRBA and incubating at 35°C for 24 h. Colonies were measured using a colony counter (Model: Scan 300, Inter science). The count was calculated as log colony-forming units per gram.

### Determination of microbial load

Plates containing distinct colonies were selected and counted using a colony counter (Model: Scan 300, Inter science). Then, the microbial load was calculated using the method and using Equation 1 (19).

(1) $$N=\frac{\sum n}{S\times d}$$

where N = total number of bacteria, yeast, mold, lactic acid bacteria (cfu/g) of the sample, *n* = average number of bacterial colonies from different dilutions containing 30–300 colonies, *S* = volume of sample for plating (ml), and *d* = dilution factor of the *Awaze* paste sample taken from the first count that should be included from 30 to 300 colonies.

### Data analysis

Statistical analysis was conducted using SPSS software, Version 24 (IBM, Chicago, IL, United States). The experiments were planed using factorial completely randomized design with three replications using SPSS software, Version 24. Tukey multiple range comparison test were utilized to investigate significant differences between the treatments, with a 95% confidence interval at *p* < 0.05. A three factor experimental design was employed. Minitab software, Version 17, was employed to examine the interaction effect between packaging material, storage temperature, and storage time.

## Results and discussion

### Physical and color value (a*) change

**Figure 1:**
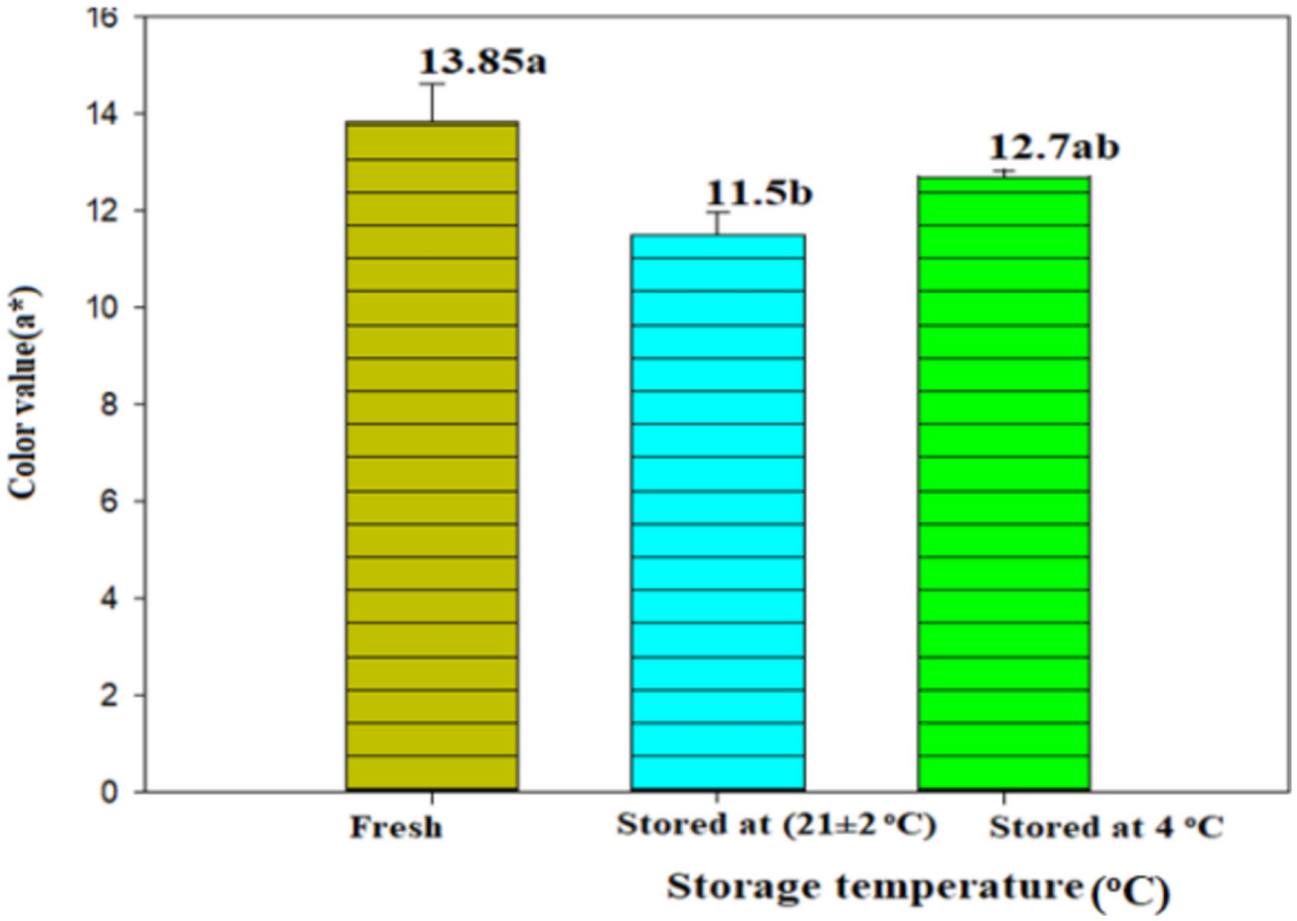
Color value (a*) of *Awaze* paste stored in glass bottle at room temperature and refrigeration temperature for 300 days of storage time.

*Awaze* paste stored in *Shekella* pots, Gourds (*Qelle*), plastic bags, plastic bottles, glass bottles, and metal can showed physical changes during the 300-day storage period, as shown in Table 1. Upon physical observation of the stored *Awaze* paste, mold growth was evident after 120, 180, 240, and 300 days on samples stored in atmospheric conditions using *Shekella* pots, plastic bags, Gourds (*Qelle*), and glass bottles, respectively. A color change from red to dark was noted in samples stored in metal cans after 180 days of storage. Samples stored in Gourds (*Qelle*) solidified after 240 days, likely due to water absorption by the packaging material. However, samples stored at refrigeration temperature, except for those in metal cans, exhibited no physical changes after 300 days. A color change was observed after 240 days in samples stored in metal cans at refrigeration temperature (Table 1). For sample, for production a glass bottle, the lowest color value (a* = 11.5) was recorded in samples stored at atmospheric temperature (room temperature), while those stored at refrigeration temperature had the highest color value (a* = 12.7) with the least change compared to fresh *Awaze* (Figure 1). In general, *Awaze* paste stored at refrigeration temperature preserved color value (a*) better than those stored at room temperature.

**Table 1:** Physical change of *Awaze* paste stored in different storage material and temperature at different time.

Temperature	Storage material	Physical change					
		Time (days)					
		30	60	120	180	240	300
Room temperature (21 ± 2°C)	*Shekella (traditional)*	N	N	N	Mold grow	Mold grow	Mold grow
	*Qelle (traditional)*	N	N	N	N	Mold grow	Mold grow
	Plastic bag	N	N	N	Mold grow, texture change	Mold grow, texture change	Mold grow, texture change
	Plastic bottle	N	N	Mold grow	Mold grow	mold grow	Mold grow
	Glass bottle	N	N	N	N	N	Mold grow
	Metal can	N	N	N	Color change	Color change	Color change
Refrigeration temperature (4°C)	*Shekella (traditional)*	N	N	N	N	N	N
	*Gourd (Qelle)*	N	N	N	N	N	N
	Plastic bag	N	N	N	N	N	N
	Plastic bottle	N	N	N	N	N	N
	Glass bottle	N	N	N	N	N	N
	Metal can	N	N	N	N	Color change	Color change

N, stands for normal.

### pH value

**Figure 2:**
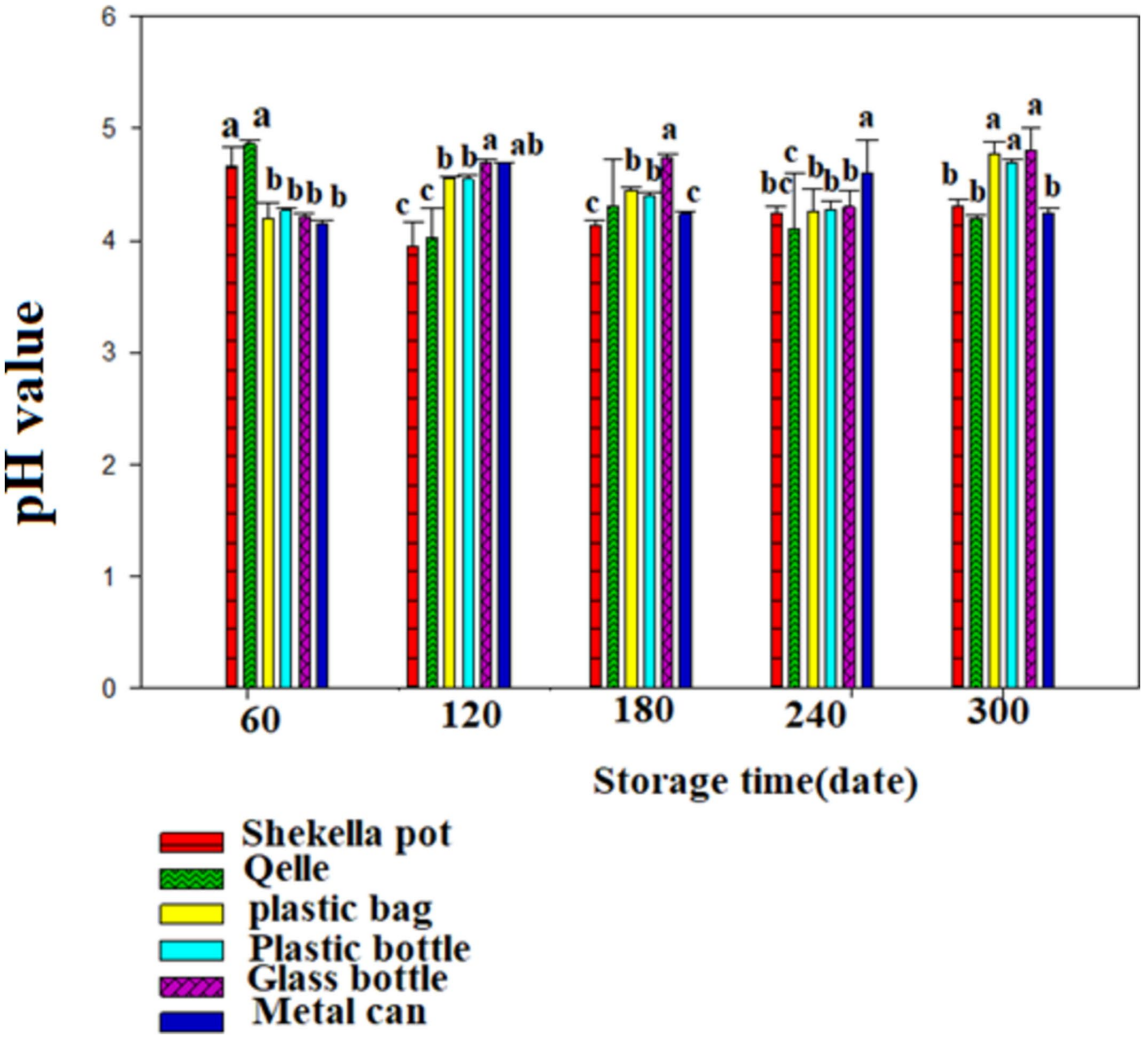
Changes in pH value during storage in the *Awaze* paste stored at different packaging material and room temperature (21 ± 2°C).

**Figure 3:**
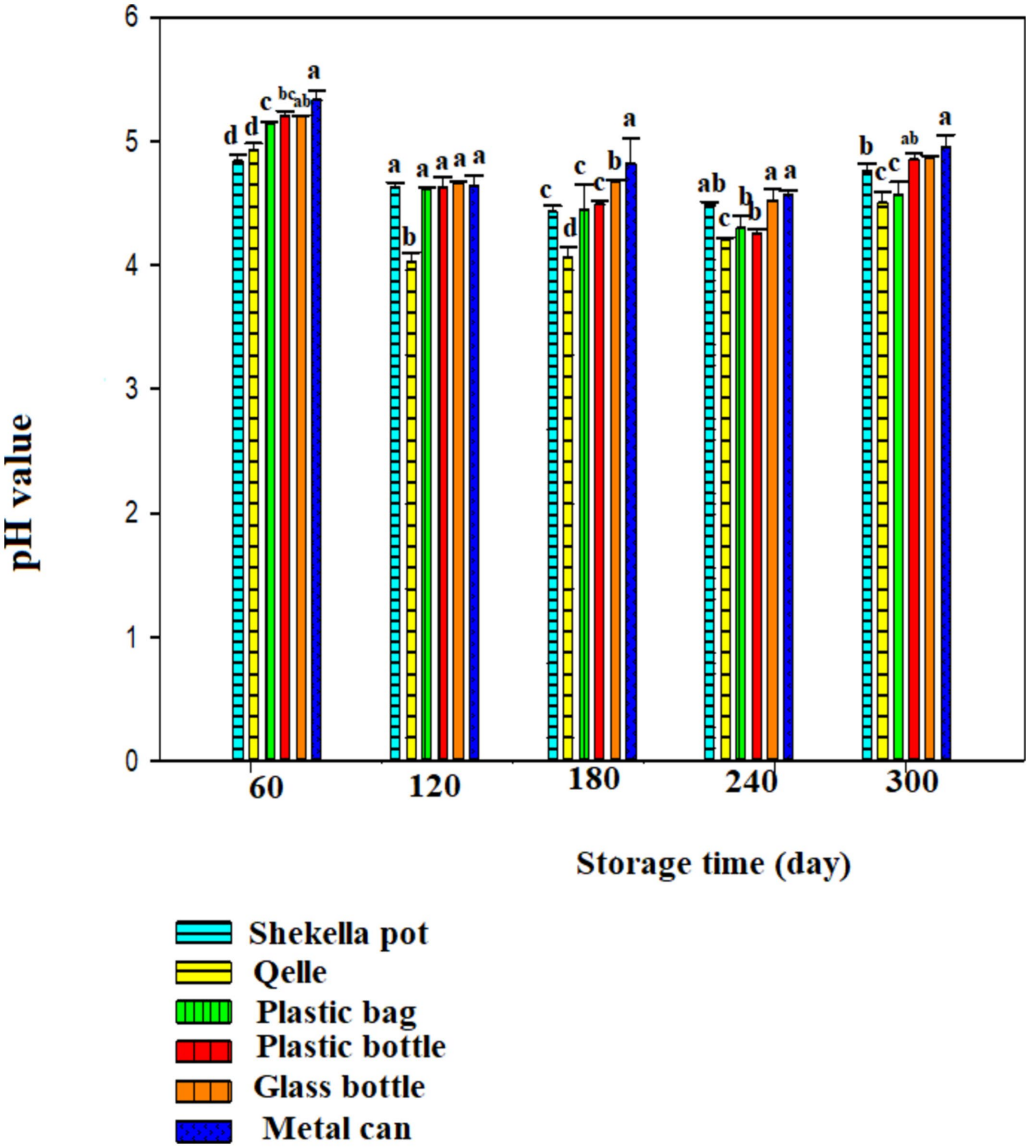
Changes in pH value during storage in the *Awaze* paste stored at different packaging material and refrigeration temperature (4°C).

The pH of *Awaze* pastes during the storage time with different packaging materials is presented in Figures 2, 3. The initial pH value of fresh *Awaze* paste was 5.25. The pH values decreased significantly (*p* < 0.05) during the storage time under both room temperature (21 ± 2°C) and refrigeration temperature (4°C) conditions, for all packaging materials. The pH values higher than 4.6 grouped as low acidic food and below 4.6 grouped as acidic food (20). The decline in pH value was more noticeable for pastes stored in atmospheric condition than the refrigeration condition. In both storage scenarios, the lowest pH values after 300 days of storage were observed in the *Qelle* package (4.2 and 4.5 for atmospheric and refrigerated storage conditions, respectively). A previous study on hot pepper paste reported pH values ranging from 3.8 to 8.79 (21), while the pH values of the sweet red pepper paste were found to be between 4.35 and 5.42 (10). These findings align with the results of this study. Generally, the pH value change at different packaging and temperature conditions indicated that pH value reduced to the level of either low acidic food or acidic food pH value during storage time.

### Titratable acidity

**Figure 4:**
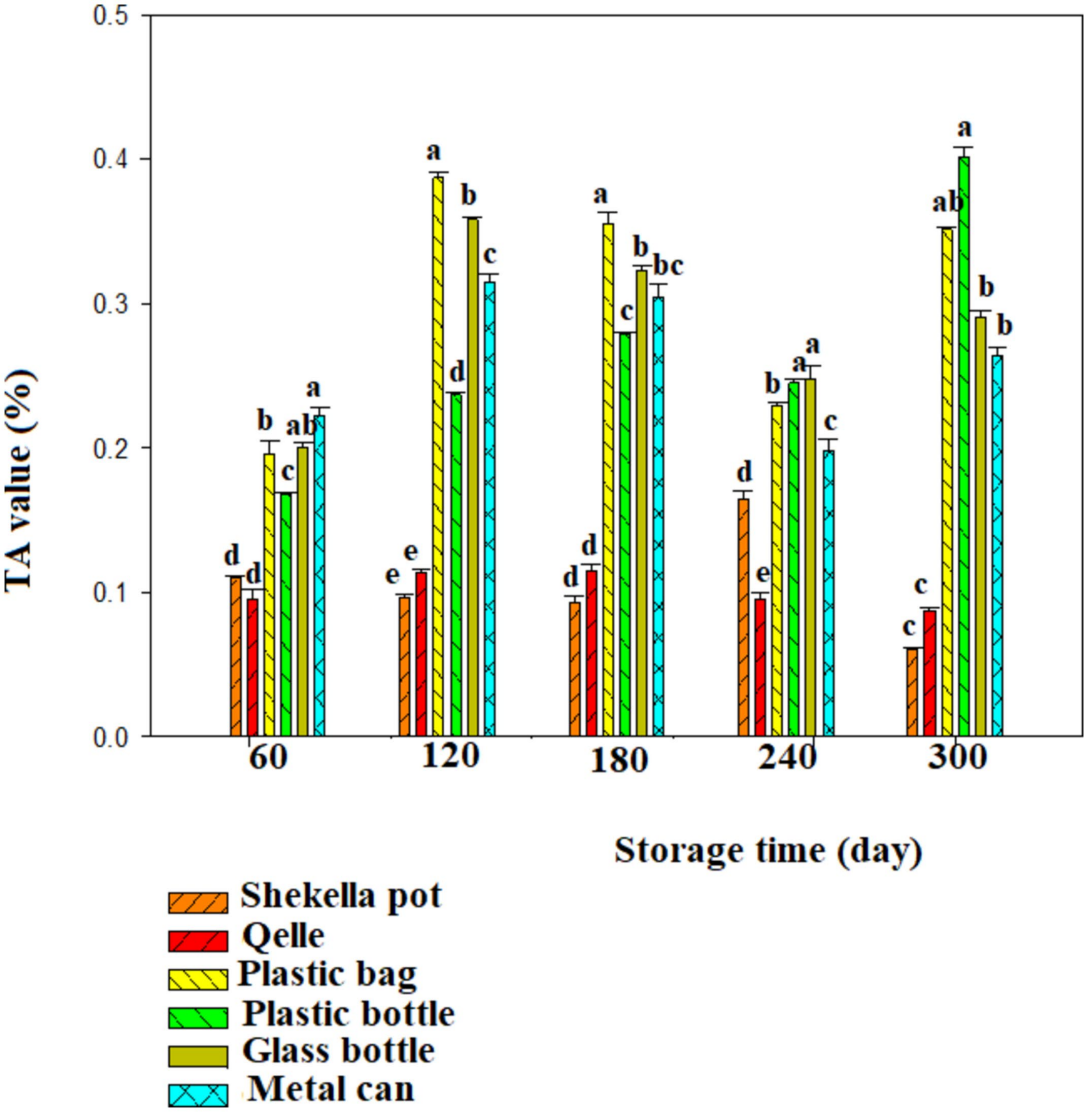
Changes in titratable acidity during storage in the *Awaze* paste stored at different packaging material and room temperature (21 ± 2°C).

**Figure 5:**
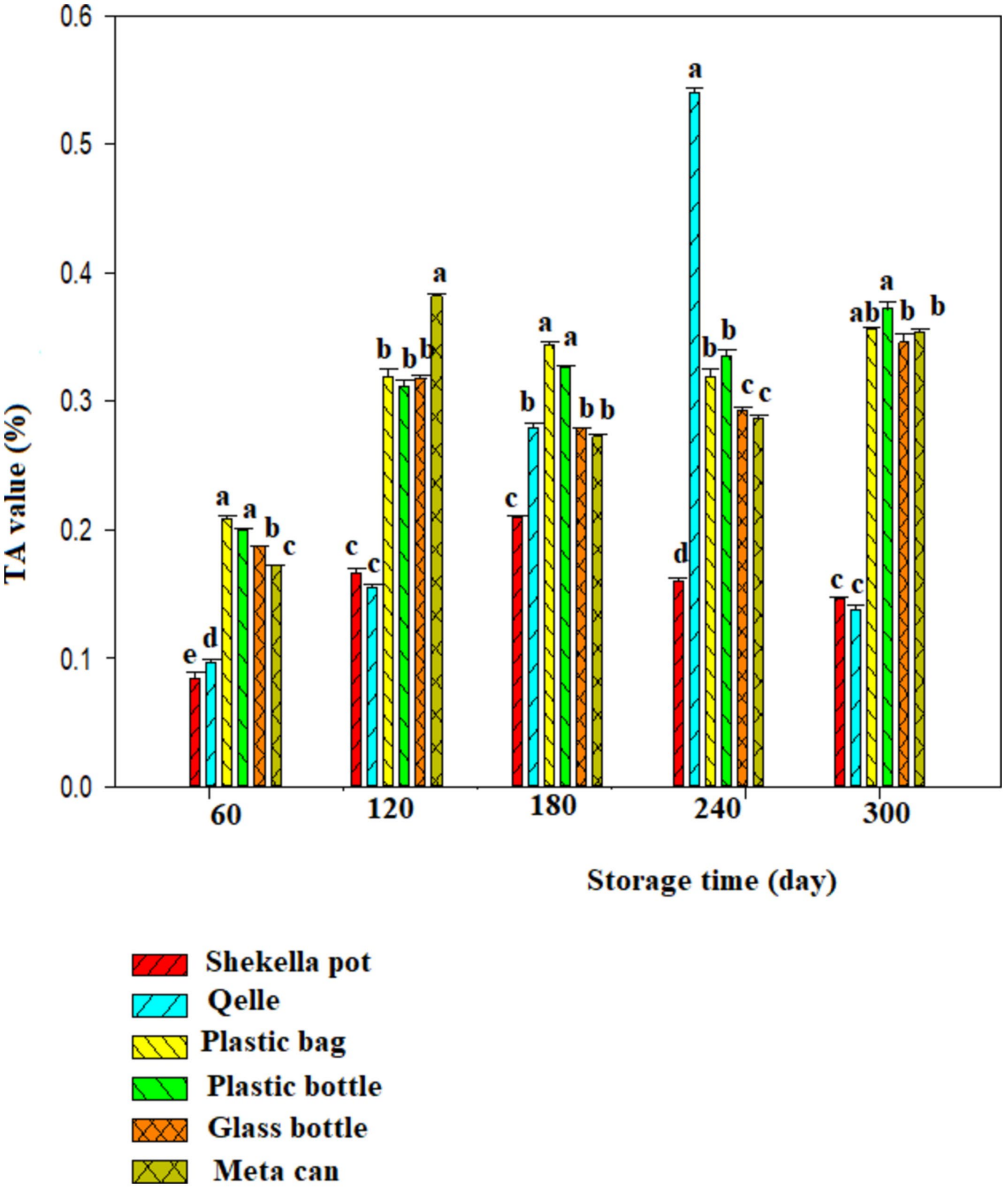
Changes in titratable acidity during storage in the *Awaze* paste stored at different packaging material and refrigeration temperature (4°C).

Figures 4, 5 presents the titratable acidity levels of *Awaze* paste stored in different types of packaging materials and storage condition. Samples stored at atmospheric condition (21 ± 2°C) in different packaging methods had TA value ranging from 0.06 to 0.387% throughout the storage period. In contrast, samples stored at refrigeration temperature (4°C) had TA value ranging from 0.085 to 0.54%. Samples packed in *Shekella* pot and *Qelle* packaging exhibited relatively low TA value, while samples stored in plastic bottles, glass bottles, and metal cans showed higher TA value. This difference could be attributed to variations in oxygen levels from diffusion through the packaging, as well as the difference in the filling and packaging procedures that may lead to oxidation of the *Awaze* paste. Generally, higher TA value was observed during refrigerated storage conditions. A previous study reported that TA values for sweet red pepper paste ranging from 0.25 to 0.98 (10), which are higher than the results obtained in the current study. This difference could be due to variations in ingredient composition, storage temperature, packaging materials, and storage duration. Generally, TA value of *Awaze* paste during storage time (300 days) indicate significant variation in the quality of the paste.

### Microbial stability of *Awaze* paste

**Figure 6:**
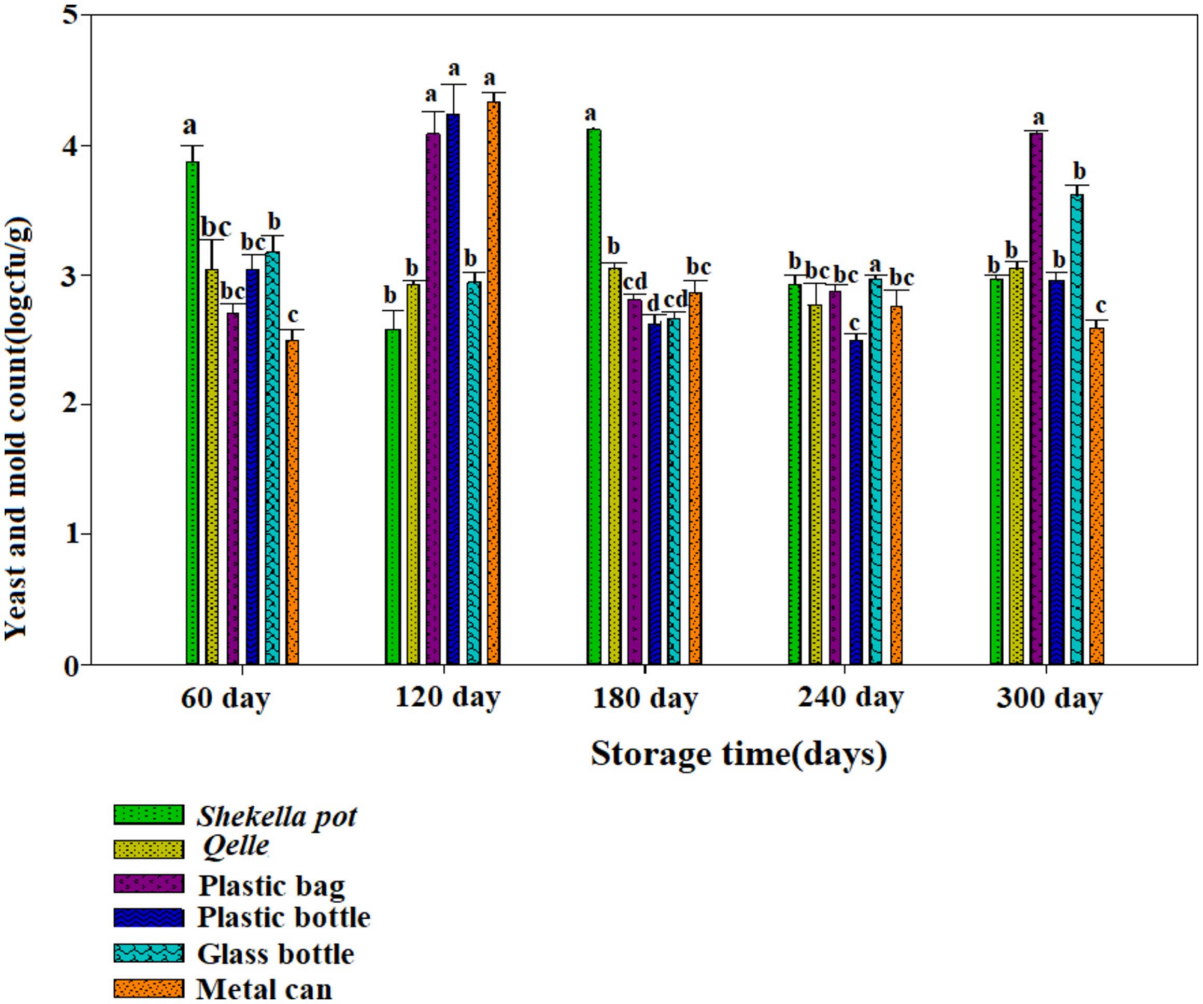
Changes in yeast and mold counts during storage in the *Awaze* paste stored at different packaging material and room temperature (21 ± 2°C).

**Figure 7:**
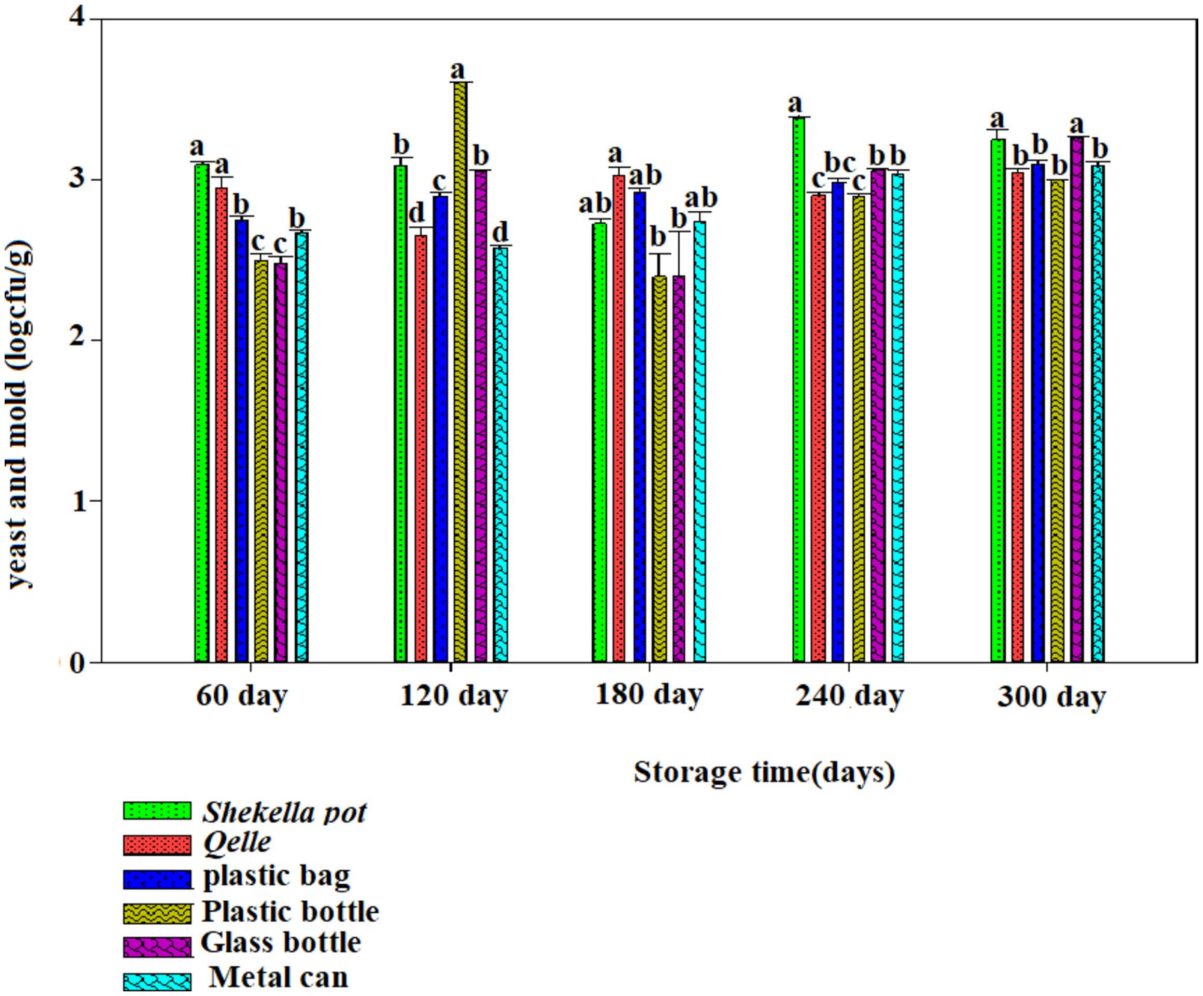
Changes in yeast and mold counts during storage in the *Awaze* paste stored at different packaging material and refrigeration temperature (4°C).

**Figure 8:**
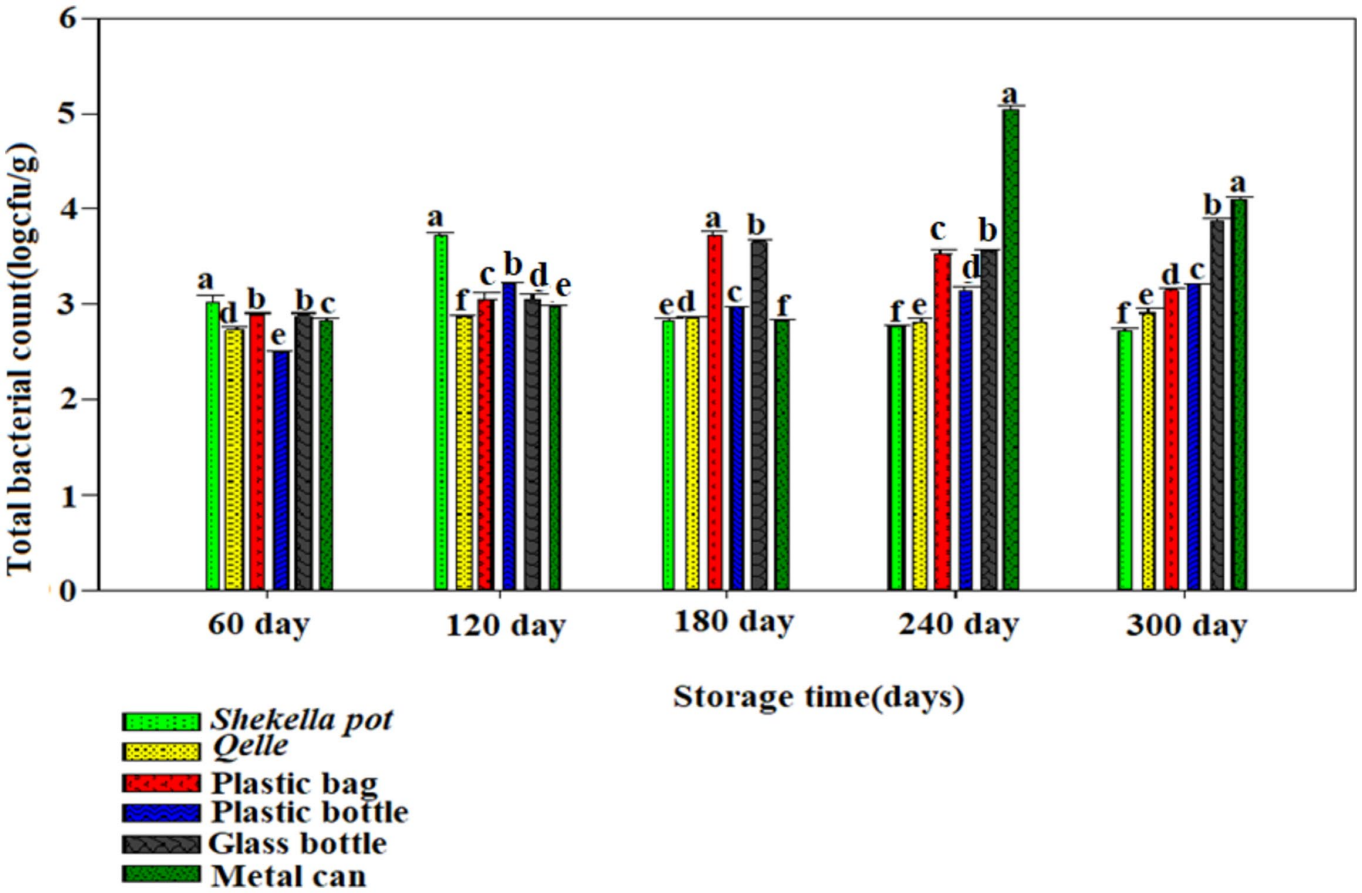
Changes in TBC during storage in *Awaze* paste stored at different packaging material and room temperature (21 ± 2°C).

**Figure 9:**
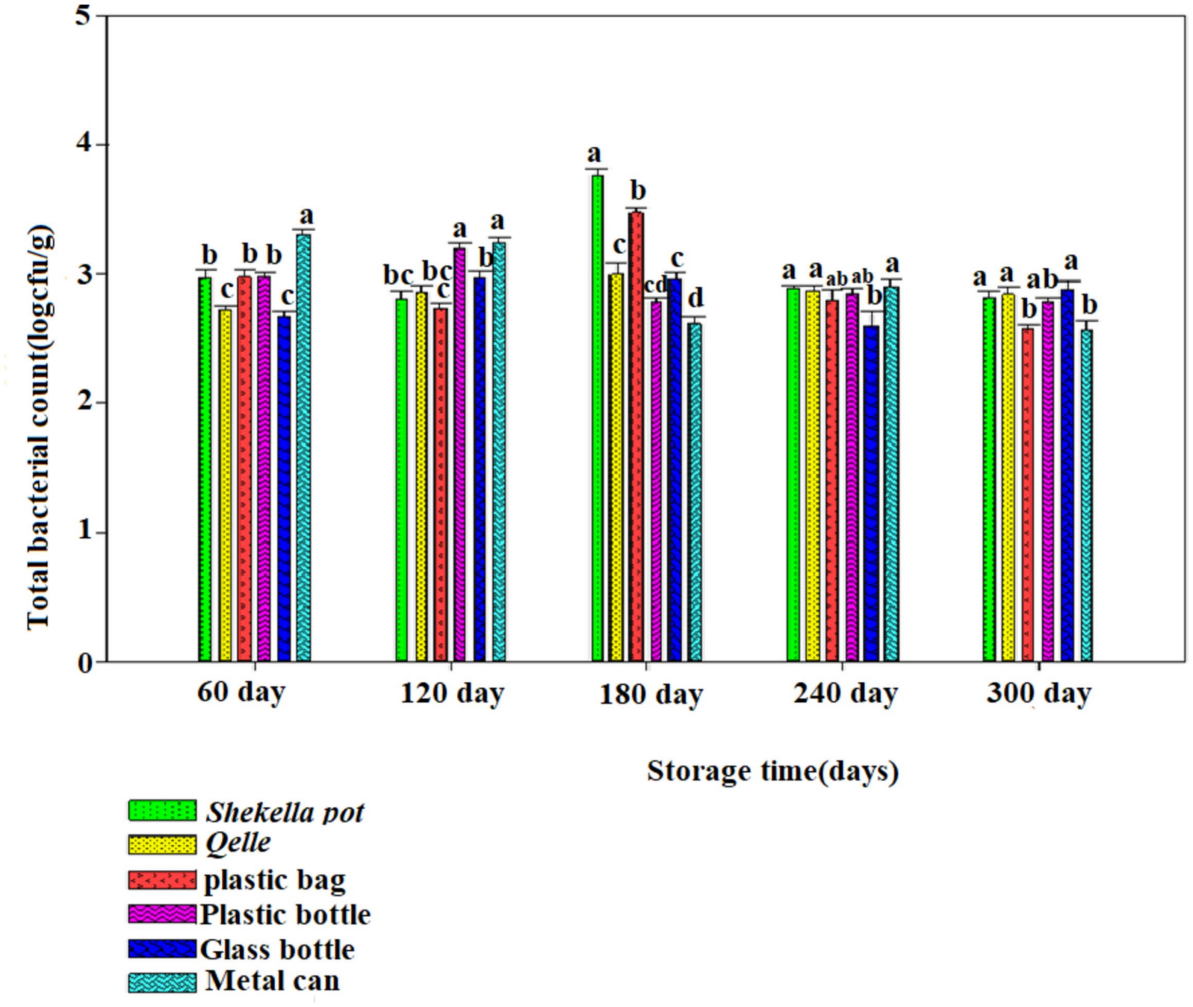
Changes in TBC during storage in *Awaze* paste stored at different packaging material and refrigeration temperature (4°C).

**Figure 10:**
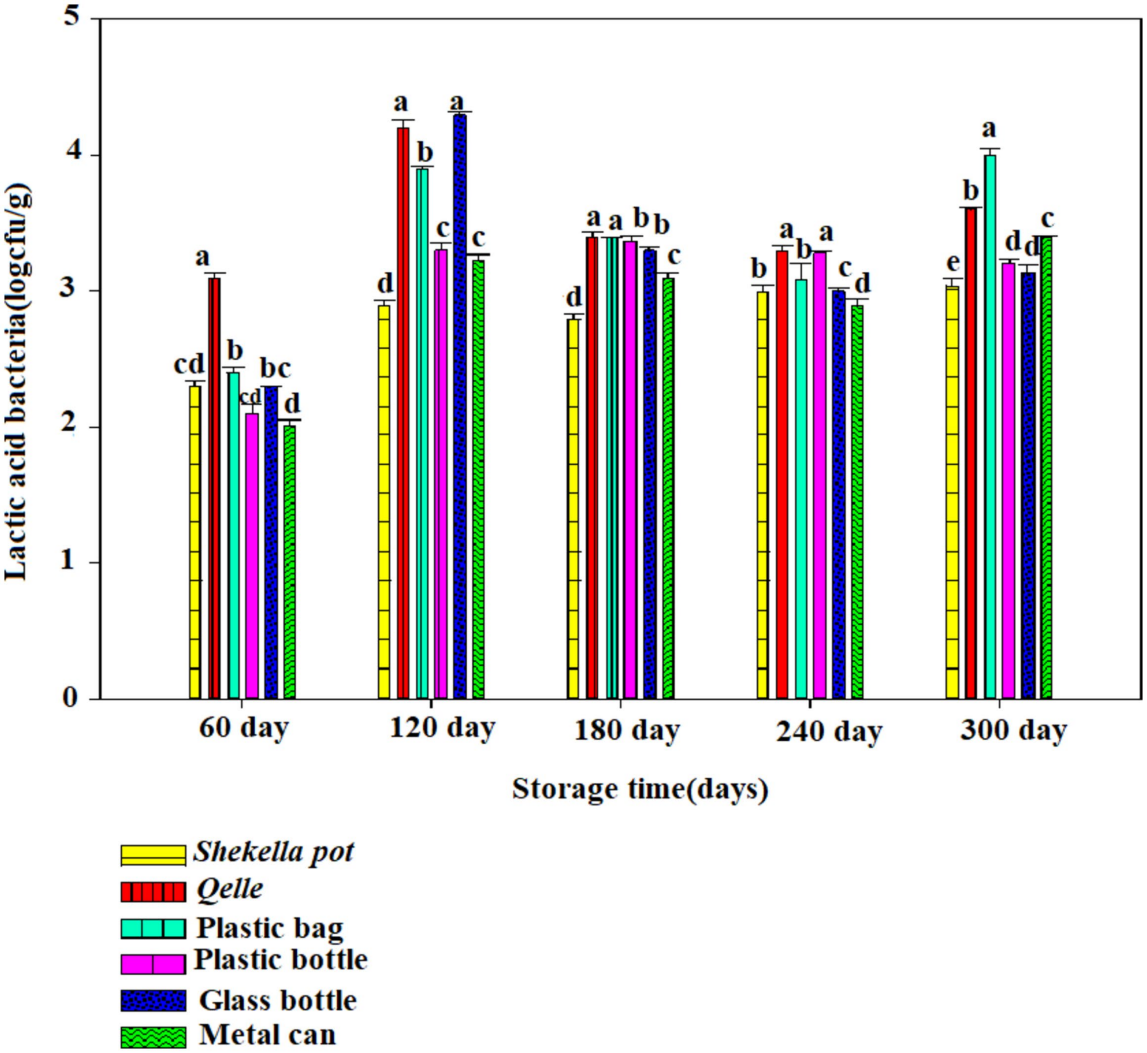
Changes in LAB during storage in *Awaze* paste stored by different packaging materials and at room temperature (21 ± 2°C).

**Figure 11:**
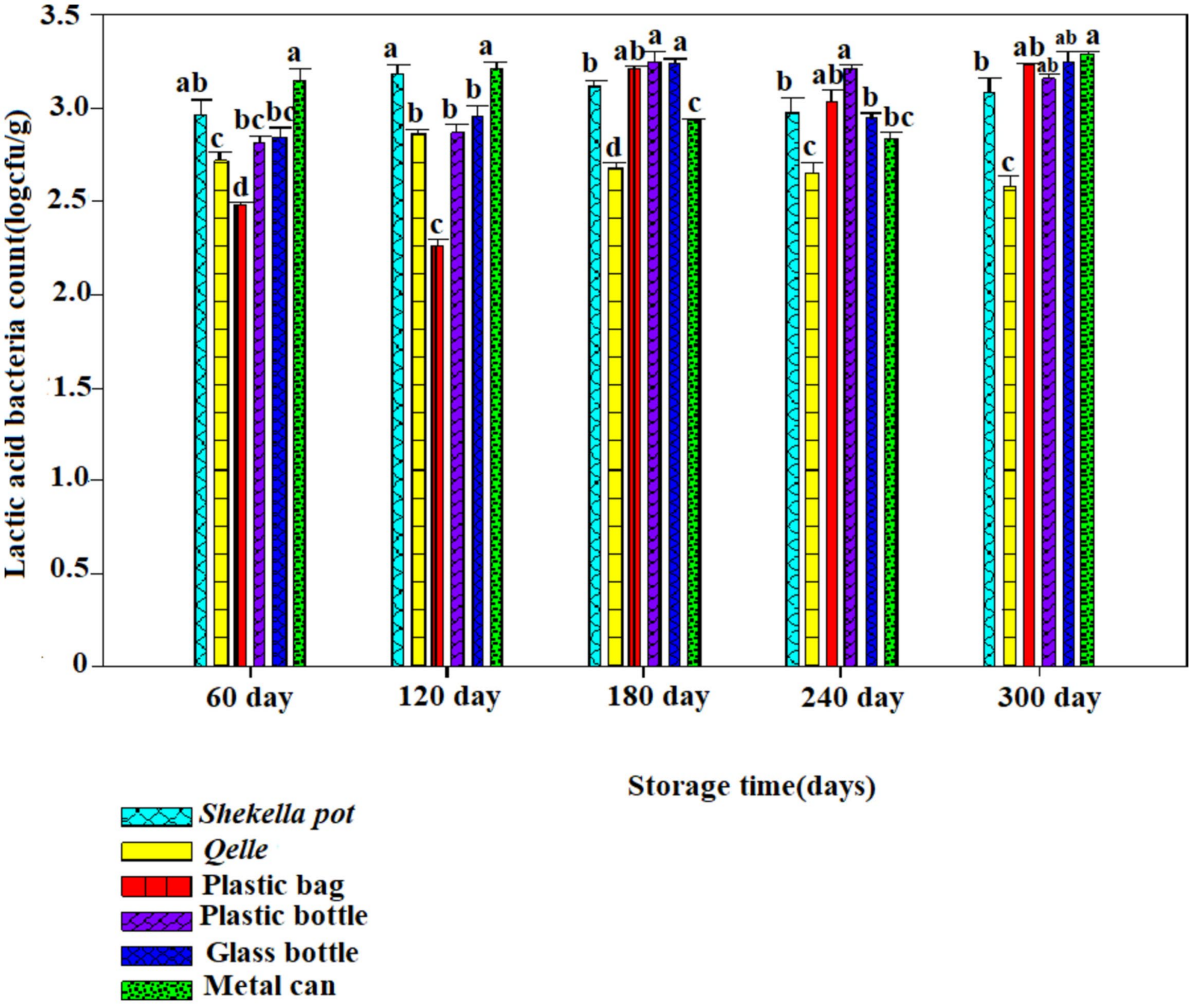
Changes in LAB during storage in *Awaze* paste stored by different packaging materials and at refrigeration temperature (4°C).

**Figure 12:**
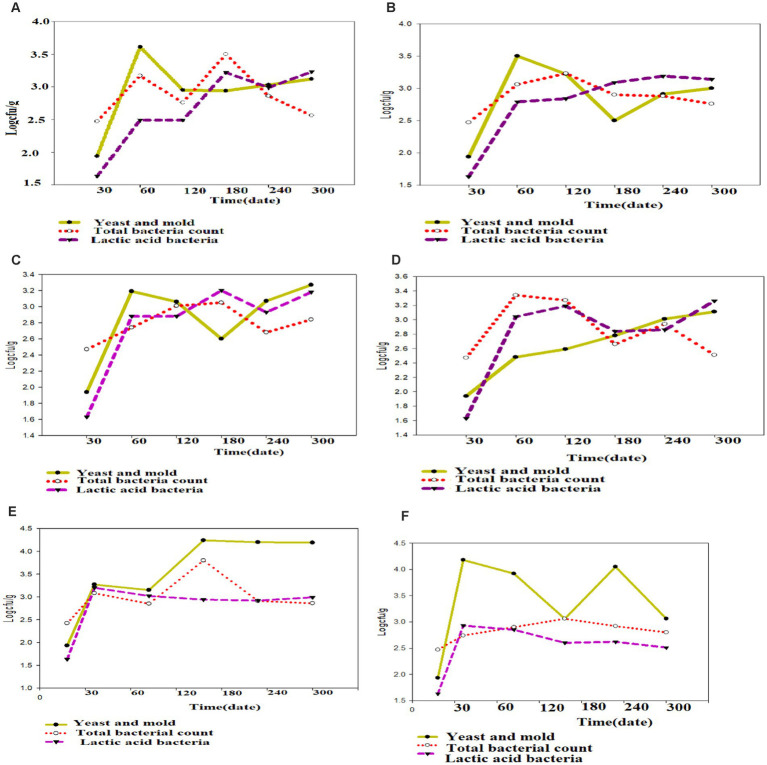
Growth pattern of yeast and mold, TBC and LAB during *Awaze* paste storage in **(A)** plastic bag, **(B)** plastic bottle, **(C)** glass bottle, **(D)** metal can, **(E)** *Shekella* pot, **(F)** *Gourd (Qelle)* at refrigeration temperature (4°C).

The microbiological analysis revealed that coliforms were not detected in fresh *Awaze* or in the final product at any point during the storage period. However, yeast, mold, total bacterial count (TBC), and lactic acid bacteria (LAB) were observed starting from the first month of storage and continued to be present thereafter (Figures 6–12).

### Yeast and mold count

Figures 6, 7 illustrate the yeast and mold counts of *Awaze* paste over the storage period. The initial yeast and mold values of fresh *Awaze* paste was 1.93 log cfu/g. The counts increased significantly (*p* < 0.05) across all packaging materials and storage conditions as the storage period progressed. Mold can thrive in the paste due to its water activity, and it can grow even under low moisture, low temperature, and high salt conditions (22). The highest (*p* < 0.05) yeast and mold counts were observed in plastic bags, plastic bottles, and metal cans at 4.08, 4.23, and 4.32 logcfu/g, respectively, after one hundred and twenty (120) days of storage at room temperature (21 ± 2°C), slightly above the acceptable limit set by the international food standard. According to the International Commission for the Microbiological Specifications for Foods (ICMSF), yeast and mold counts should be lower than 4 log cfu/g for food samples (19).

Considering 4 logcfu/g as the acceptable limit for molds and yeast, samples stored in plastic bags, plastic bottles, and metal cans were not acceptable after one hundred twenty (120) days of storage at room temperature (21 ± 2°C). *Awaze* paste samples stored in Gourds (*Qelle*) and glass bottles showed yeast and mold counts below the limit until 300 days of storage at room temperature (21 ± 2°C). At this storage condition, there was a noticeable decrease in yeast and mold count to a safe value after the peaks, possibly due to the depletion of oxygen and the accumulation of carbon dioxide as a result of yeast and mold respiration. Samples stored in various packages at atmospheric temperature (21 ± 2°C) exceeded the acceptable yeast and mold count limit of 4 log cfu/g. However, for all packaging materials kept at refrigeration temperature (4°C) for 300 days, the yeast and mold counts remained within the acceptable limit (Figures 6, 7).

Yeast strains such as, *Candida versatilis, Zygosaccharmycesrouxii*, and *C. etchellsii* produce gas during the fermentation of pastes and grow when oxygen levels increase (23). More permeable film packaging retained higher yeast and mold counts than less permeable packaging materials. *Awaze* paste stored in glass bottles had lower yeast and mold counts compared to *Shekella* pots, Gourds (*Qelle*), plastic bags, plastic bottles, and metal cans, possibly due to the relatively low oxygen permeability of glass bottles. Similarly, *Awaze* paste stored in Gourds (*Qelle*) also had low yeast and mold counts, likely due to the low permeability of the storage material. A previous study on sweet red pepper paste with different packaging materials (PET and PP) found yeast and mold counts of 3.3 log cfu/g (10), that is in consistent with this study. Korean red pepper paste packed under modified atmosphere with high barrier plastic film at 13°C had yeast and mold counts of 6.5 log cfu/g (9), higher than the results of the current study. In general, *Awaze* paste stored at room temperature (21 ± 2°C) in all packaging materials had higher yeast and mold counts compared to refrigeration temperature (4°C). Glass bottles and Gourds (*Qelle*) had the lowest yeast and mold counts (within the acceptable limit) compared to *Shekella* pots, plastic bags, plastic bottles, and metal cans in both storage temperature conditions (21 ± 2°C and 4°C).

### Total bacterial plate count

The TBC of *Awaze* paste is presented in Figures 8, 9. The TBC of *Awaze* paste remains relatively stable during storage. The initial total bacteria count of fresh *Awaze* paste was 2.47 log cfu/g. Low moisture, low temperature, and high salt levels are unfavorable for bacterial growth. The acceptable limit for the TBC is 5 log cfu/g for spices and foods that require further cooking before consumption (24). It was determined that the paste samples stored in *Shekella* pot, *Gourd (Qelle)*, plastic bag, and glass bottle were within the acceptable limit at atmospheric temperature (21 ± 2°C). The *Awaze* paste sample packed in a metal can showed a slightly higher TBC (5.02 log cfu/g) after 240 days of storage (Figure 8). After 300 days of storage, samples stored using all packaging materials at 4°C were acceptable in terms of safe bacterial levels (Figure 9). Previous studies have reported TBC ranging from 0.13 to 8.6 log cfu/g for hot red pepper paste produced using various techniques (21). Another study on Korean red pepper paste using different packaging films reported TBC in the range of 5.2 to 5.4 log cfu/g (9), which is higher than the findings of this study. Overall, our data show that samples stored in all packaging materials under refrigeration conditions had the lowest TBC, which falls within acceptable food standards. *Awaze* samples stored in a metal can at room temperature had the highest TBC, exceeding the acceptable limit of food standards.

### Lactic acid bacteria

There was an increase in LAB with storage period. The initial lactic acid bacteria count of fresh *Awaze* paste was 1.63 log cfu/g. The highest value observed was 4.32 log cfu/g for glass bottle followed by *Gourd (Qelle)* (4.3 log cfu/g) stored at atmospheric temperature (21 ± 2°C) for 120 days (Figure 6), but it decreased to a value of below 3 log cfu/g as the storage proceeded to 300 days. The decrease with storage period could be due to the depletion of oxygen and accumulation of carbon dioxide due to respiration of the bacteria. For refrigerated storage, the maximum value observed was 3.25 log cfu/g for *Awaze* paste stored using a metal can for 300 days (Figures 10, 12). For all packaging materials, samples stored at room temperature (21 ± 2°C) had higher LAB compared to samples stored at refrigeration temperature (4°C). Lactic acid bacteria count at atmospheric temperature observed a maximum of 4 log cfu/g and for refrigeration temperature observed 3.25 up to 300 days storage. This indicates that the final stage of *Awaze* paste fermentation was carried out by lactic acid bacteria. This is consistent with the common phenomenon in food and beverage fermentation involving lactic acid bacteria. The lactic acid bacteria provide the acidic environment for yeast growth while the yeast provide vitamin and other growth factors (25). A similar observation was reported by Aşkin Uzel (10). A study on LAB growth on red pepper paste stored at 29°C using back sloping fermentation reported a value in the range of 3.5–9 log cfu/g (26), which is higher than the current result. This could be due to the back sloping fermentation process of red pepper paste that could increase the LAB. A previous study on chili pepper paste found LAB in the range of 2–8 log cfu/g (27). Another report on *Awaze* stored at atmospheric conditions reported LAB as high as 9 log cfu/g (7), this could be due to the difference in formulation and packaging.

Regarding the microbial growth pattern, some growth was observed in yeast and mold count, total bacteria count, and lactic acid bacteria in all packaging materials after 120 days stored at room temperature (21 ± 2 4°C) and 60 days stored at refrigeration temperature (4°C) after the first and second analysis. In all packaging materials except metal can, yeast and mold and lactic acid bacteria showed a similar growth pattern at 4°C storage condition. For all packaging materials that were stored at 4°C, the highest microbial growth rate was observed during the initial storage period (Figure 12). For the product that was packed using plastic bag, plastic bottle, glass bottle and metal can, the maximum microbial growth rate was during the first 60 days of the storage period of the product. Whereas for the product that was packed using *Shekella* pot and *Gourd (Qelle)*, the maximum growth rate was observed during the first 30 days storage period. Lactic acid bacteria increased steadily until they reached their maximum count of 4.45 log cfu/g when stored at room temperature (21 ± 2°C) and 3.26 log cfu/g when stored at refrigeration temperature (4°C). Generally, after 300 days of storage in all packaging materials at refrigeration temperature (4°C), yeast and mold and lactic acid bacteria increase, while total bacterial count decreases (Figure 12). The current result are in agreement with the report of Hassen et al. (7).

### Main and interaction effects of packaging material, storage temperature, and storage time for *Awaze* paste

The main and interaction effects of packaging material, storage time, and storage temperature are detailed in Table 2. Storage time significantly (*p* < 0.05) influenced the physicochemical and microbiological quality of *Awaze* paste. Packaging materials significantly (*p* < 0.05) affected the pH and acidity of the paste. Storage temperature had a significant (*p* < 0.05) impact on the pH, titratable acidity, and total bacterial count (TBC) of *Awaze* paste. The interaction between packaging material and storage time significantly (*p* < 0.05) influenced the Titratable acidity of the paste. Additionally, the interaction between storage time and storage temperature significantly (*p* < 0.05) affected both the pH and TBC of *Awaze* paste. Generally, the pH value, TA, yeast and mold, TBC, and lactic acid bacteria of *Awaze* paste was affected by storage time.

**Table 2:** Main effects and interaction effect of packaging material, storage temperature and storage time for *Awaze* paste stored at different packaging material and temperature conditions.

Parameter	(*p* value < 0.05)					
	Storage material	Storage time	Temperature	SM × ST	SM × temp	ST × temp
pH value	0.02	0.00	0.00	0.565	0.346	0.003
Titratable acidity	0.00	0.00	0.005	0.040	0.150	0.069
Yeast and mold	0.106	0.00	0.745	0.336	0.383	0.315
Total bacterial count	0.251	0.00	0.013	0.225	0.146	0.031
Lactic acid bacteria	0.564	0.00	1.00	0.241	0.263	0.059

SM, storage material; ST, storage time; temp, temperature.

## Conclusion

The study investigated the effects of packaging material type, storage temperature, and storage time on the physicochemical and microbial properties of *Awaze* paste. Significant differences in microbial load were found among various packaging materials, storage durations, and temperatures. Color changes occurred during storage at both atmospheric and refrigeration temperatures, with more pronounced color degradation at room temperature. Yeast, mold, and lactic acid bacteria counts increased over time. Storage time significantly influenced the physicochemical and microbial quality of *Awaze* paste. The interaction between packaging material type and storage time significantly affected the paste’s titratable acidity. Additionally, the interaction between storage time and temperature affected the pH and total bacterial count. The results of this study indicated that *Awaze* paste stored in a glass bottle at refrigeration temperature maintained good quality and safety. In conclusion, based on observations of physical changes, pH values, titratable acidity, yeast and mold counts, and total bacterial counts, it is safe to store *Awaze* paste in a glass bottle at 4°C for up to 300 days. Future research on modified atmospheric packaging of *Awaze* paste with well-controlled storage conditions, including relative humidity, is highly recommended.

## Data Availability

The raw data supporting the conclusions of this article will be made available by the authors, without undue reservation.

## References

[1] Kefale B Admasu M Solomon D Fanta W Abate S. Optimization of spicy red pepper paste (awaze) formulation by D-optimal mixture design. Food Sci Nutr. (2023) 12:1–16. doi: 10.1002/fsn3.3874 PMC10916596 38455199

[2] Tsegaye M Ephraim E Ashenafi M. Behaviour of *Escherichia coli* O157: H7 during the fermentation of Datta and awaze, traditional Ethiopian fermented condiments, and during product storage at ambient and refrigeration temperatures. Food Microbiol. (2004) 21:743–51. doi: 10.1016/j.fm.2004.02.003

[3] Kefale B. Review on onion drying and effect of drying on nutritional content of onion processed products. J Public Health Nutr. (2022) 5:1–8. doi: 10.35841/aajphn-5.7.134

[4] Kefale B Admasu M Workneh S Abate S. Optimization of awaze paste formulations: the effects of using spices through a mixture design approach. Heliyon. (2024) 10:e35141. doi: 10.1016/j.heliyon.2024.e35141, PMID: 39170444 PMC11336441

[5] Kefale B Delele MA Fanta SW Abate SM. Nutritional, physicochemical, functional, and textural properties of red pepper (*Capsicum annuum* L.), red onion (*Allium cepa*), ginger (*Zingiber officinale*), and garlic (*Allium sativum*): Main ingredients for the preparation of spicy foods in Ethiopia. J Food Qual. (2023) 2023:3916692. doi: 10.1155/2023/3916692

[6] Woldemariam HW Emire SA Teshome G Toepfl S Aganovic K. Physicochemical, functional, oxidative stability and rheological properties of red pepper (*Capsicum annuum* L.) powder and paste. Int J Food Prop. (2021) 24:1416–37. doi: 10.1080/10942912.2021.1969945

[7] Idris A Mehari T Ashenafi M. Some microbiological and biochemical studies on the fermentation of “awaze” and “datta”, traditional Ethiopian condiments. Int J Food Sci Nutr. (2001) 52:5–14. doi: 10.1080/09637480020027174, PMID: 11225177

[8] Tigu F Assefa F Mehari T Ashenafi M. Probiotic property of lactic acid bacteria from traditional fermented condiments: Datta and awaze. Int Food Res J. (2016) 23:770–6.

[9] Lee DS Jang JD Hwang YI. The effects of using packaging films with different permeabilities on the quality of Korean fermented red pepper paste. Int J Food Sci Technol. (2002) 37:255–61. doi: 10.1046/j.1365-2621.2002.00564.x

[10] Aşkin Uzel R. Preservation of sweet red pepper paste quality: effect of packing material, ozone gas and protective agent use. Food Sci Technol. (2018) 38:698–703. doi: 10.1590/1678-457x.13917

[11] Byun KH Cho MJ Park SY Chun HS Do Ha S. Effects of gamma ray, electron beam, and X-ray on the reduction of aspergillus flavus on red pepper powder (*Capsicum annuum* L.) and gochujang (red pepper paste). Food Sci Technol Int. (2019) 25:649–58. doi: 10.1177/1082013219857019, PMID: 31213080

[12] Păucean A Kádár CB Simon E Vodnar DC Ranga F Rusu IE. Freeze-Dried Powder of Fermented Chili Paste—New Approach to Cured Salami Production. Foods. (2022) 11:3716. doi: 10.3390/foods11223716 36429308 PMC9689597

[13] Il Cho W Yi JY Chung MS. Pasteurization of fermented red pepper paste by ohmic heating. Innov Food Sci Emerg Technol. (2016) 34:180–6. doi: 10.1016/j.ifset.2016.01.015

[14] Titus S Wojtek D. “Spices sector in Ethiopia,” (2020) 1–16.

[15] Terrones-Fernandez I Casino P López A Peiró S Ríos S Nardi-Ricart A. Improvement of the pour plate method by separate sterilization of agar and other medium components and reduction of the agar concentration. Microbiol Spectr. (2023) 11:1–12. doi: 10.1128/spectrum.03161-22 PMC9927588 36625633

[16] Pathare PB Opara UL Al-Said FAJ. Colour measurement and analysis in fresh and processed foods: a review. Food Bioprocess Technol. (2013) 6:36–60. doi: 10.1007/s11947-012-0867-9

[17] Choi JE Lee JH. Non-Newtonian characteristics of gochujang and chogochujang at different temperatures. Prev Nutr Food Sci. (2017) 22:62–6. doi: 10.3746/pnf.2017.22.1.62, PMID: 28401090 PMC5383144

[18] AOAC. Association of official analytical chemists. (2003) 1:73–80.

[19] ICMSF. International Comission on Microbiological Specifications for Foods (2010) Ensuring Glob. Food Saf. 91–8.

[20] Ramalingam S Bahuguna A Lim S Joe AR Lee JS Kim SY. Physicochemical, microbial, and volatile compound characteristics of gochujang, fermented red pepper paste, produced by traditional cottage industries. Foods. (2022) 11:375. doi: 10.3390/foods11030375 35159525 PMC8834593

[21] Erkmen O. Effects of production techniques on the quality of hot pepper paste. J Food Eng. (2004) 64:173–8. doi: 10.1016/j.jfoodeng.2003.09.028

[22] Devi TB Dash SK Bal LM Sahoo NR. Physicochemical and microbiological characteristics of ginger paste (cv. Suprabha) during storage in different packaging and temperature conditions physicochemical and microbiological characteristics of ginger paste (cv. Suprabha) during storage in. Cogent Food Agric. (2016) 16:1223261. doi: 10.1080/23311932.2016.1223261

[23] Hong SI Park WS. Use of color indicators as an active packaging system for evaluating kimchi fermentation. J Food Eng. (2000) 46:67–72. doi: 10.1016/S0308-8146(00)00141-2

[24] Topno PN Vinothini Jayaprakash SH Varadaiah V Sheshagiri SH Srinivas PM. Ginger-garlic paste in retort pouches and its quality. J Food Process Eng. (2013) 36:1–8. doi: 10.1111/j.1745-4530.2011.00645.x

[25] Oyeyiola GP. Fermentation of millet to produce kamu a Nigerian starch-cake food. World Journal of Microbiology and Biotechnology (1991) 7:196–201. doi: 10.1007/BF00328990 24424932

[26] Rita A. Optimization of lactic acid fermentation in the production of red pepper paste. (2017) 1–68.

[27] Vegas C Zavaleta AI Zarzoso B. Optimization of fermentation process conditions for chili pepper (*Capsicum frutescens*) fruit using response surface methodology. Agron Colomb. (2018) 36:88–96. doi: 10.15446/agron.colomb.v36n1.69164

